# lncRNA CASC2 Enhances ^131^I Sensitivity in Papillary Thyroid Cancer by Sponging miR-155

**DOI:** 10.1155/2020/7183629

**Published:** 2020-10-19

**Authors:** Ling Tao, Ping Tian, Li Yang, Xiangyang Guo

**Affiliations:** ^1^Institute of Inspection Technology, Xinyang Vocational and Technical College, Key Laboratory of Geriatric Diseases of Xinyang, Xinyang 464000, China; ^2^Department of Endocrinology, Xinyang Central Hospital, Xinyang 464000, China

## Abstract

Long noncoding RNA cancer susceptibility candidate 2 (CASC2) has been reported to play an anticancer role in papillary thyroid cancer (PTC). Radioiodine (^131^I) is a common option for the treatment of PTC. However, the role and mechanism of CASC2 in ^131^I sensitivity remain unclear. In this study, ^131^I-resistant cells were constructed through continuous treatment of ^131^I. The expression levels of CASC2 and miR-155 were measured by qRT-PCR. The IC50 of ^131^I was analyzed by cell viability using MTT assay. Flow cytometry was conducted to determine cell apoptosis induced by ^131^I. The association between CASC2 and miR-155 was evaluated by luciferase assay and RNA immunoprecipitation. A mouse xenograft model was built to explore the effect of CASC2 on the growth of ^131^I-resistant PTC cells *in vivo*. Results showed that CASC2 expression was decreased in PTC tissues and cells, and low expression of CASC2 was associated with poor outcome of patients. CASC2 level was reduced in ^131^I-resistant cells. Knockdown of CASC2 inhibited ^131^I sensitivity in thyroid cancer cells. Overexpression of CASC2 enhanced ^131^I sensitivity in constructed resistant PTC cells. CASC2 was a decoy of miR-155, and CASC2-mediated promotion of ^131^I sensitivity was weakened by decreasing miR-155. Abundance of CASC2 inhibited the growth of ^131^I-resistant cells *in vivo*. As a conclusion, CASC2 increases ^131^I sensitivity in PTC by sponging miR-155, providing a novel target for the treatment of thyroid cancer patients with ^131^I resistance.

## 1. Introduction

Papillary thyroid cancer (PTC) is regarded as a low-risk thyroid cancer with high morbidity [1]. In recent years, great development has been gained on the diagnosis and treatment of thyroid cancer [2]. Radioiodine (^131^I) exposure is the standard adjuvant treatment for patients with thyroid cancer [3]. However, some patients would fail to respond to ^131^I therapy with an overall survival less than 50% due to the local recurrence and distant metastasis [4]. Noncoding RNAs are associated with PTC development and can enhance ^131^I therapeutic function and improve the survival of patients [5–7].

Noncoding RNAs, including long noncoding RNAs (lncRNAs) and microRNAs (miRNAs), play as important biomarkers by regulating cancer development and treatment in endocrine-related cancers, including thyroid cancer [8]. lncRNAs with >200 nucleotides in length and absent ability of coding proteins have been reported to play important roles in the diagnosis, prognosis, and therapeutics of thyroid cancer [9]. Moreover, lncRNA can drive radioresponse and regulate the outcomes of patients after radiotherapy [10]. For example, Liu et al. report that lncRNA maternally expressed gene 3 (MEG3) can increase ^131^I sensitivity by sponging miR-182 in thyroid cancer [6]. Moreover, Xiang et al. suggest that lncRNA solute carrier family 6 member 9 (SLC6A9) sensitizes PTC cells to ^131^I treatment [7]. Long noncoding RNA cancer susceptibility candidate 2 (CASC2) has been demonstrated as a tumor suppressor by regulating cell proliferation, apoptosis, migration, and chemoresistance in human cancers, including hepatocellular carcinoma, gastric cancer, and prostate cancer [11–13]. Furthermore, emerging evidences suggest that CASC2 is lowly expressed and inhibits cell proliferation in PTC [14, 15]. However, little is known about the role of CASC2 in ^131^I sensitivity to PTC.

In the present study, we first constructed the ^131^I-resistant cells. Moreover, we investigated the effect of CASC2 on ^131^I sensitivity to PTC cells by detecting cell viability and apoptosis. In addition, we explored the target association between CASC2 and miR-155 to elucidate the regulatory mechanism of CASC2.

## 2. Materials and Methods

### 2.1. Patients and Tissues

A total of 46 paired cancer tissues and surrounding normal samples were collected from patients with PTC during surgical resection at Xinyang Central Hospital and then stored at -80°C in the Key Laboratory of Geriatric Diseases of Xinyang until used. The clinical features of patients are displayed in Table 1. Furthermore, another 50 PTC patients (23 males and 27 females; age: 45-60 years old; 39 with lymph node metastasis and 11 without) who have received ^131^I treatment at least 1 year (200 mCi for patients with lymph node metastasis and 100 mCi for those without lymph node metastasis) prior to the study were recruited, and a follow-up study was performed for analysis of the overall survival of patients. All participants have provided the written informed consent, and this study was approved by the ethics committee of Xinyang Central Hospital.

### 2.2. Cell Culture, ^131^I-Resistant Cell Construction, and Transfection

Thyroid cancer cell lines (FTC-133, TPC-1, BCPAP, and IHH-4) and human thyroid follicular cell line Nthy-ori 3-1 cells were purchased from BeNa Culture Collection (Beijing, China). The cells were maintained at 37°C and 5% CO_2_. Cell culture medium was RPMI-1640 medium (Gibco, Grand Island, NY, USA) containing 10% fetal bovine serum and changed every three days. TPC-1 and IHH-4 cells with the lowest level of CASC2 were chosen to construct ^131^I-resistant cells (res-TPC-1 and res-IHH-4). In brief, TPC-1 and IHH-4 cells were exposed to a median-lethal dose of ^131^I for continuous passaging. After treatment for 12 h, IC50 of ^131^I radioactivity was analyzed by cell viability using MTT assay. The resistant cells were obtained after passaging for 8 generations (G) and identified using flow cytometry by avoiding apoptosis under ^131^I exposure. TPC-1 and res-TPC-1 cells were exposed to 1 mCi ^131^I for 12 h, and IHH-4 and res-IHH-4 cells were exposed to 0.5 mCi ^131^I for 12 h, followed by further study.

CASC2 overexpression vector (CASC2) was generated through inserting full-length CASC2 sequences into pcDNA3.1 vector (pcDNA) (Thermo Fisher, Wilmington, DE, USA). siRNA against CASC2 (si-CASC2) (sense: 5′-UUCUAGAAUUAGAAAGAACUC-3′, antisense: 5′-GUUCUUUCUAAUUCUAGAAUU-3′), siRNA negative control (si-NC) (sense: 5′-UCUCCGAACGUGUCACGUTT-3′, antisense: 5′-GUGACACGUUCGGAGAATT-3′), miR-155 mimic (sense: 5′-UUAAUGCUAAUUGUGAUAGGGGU-3′, antisense: 5′-CCCUAUCACAAUUAGCAUUAAUU-3′), and miRNA negative control (miR-NC) (sense: 5′-UUCCUCCGAACGUGUCACGUTT-3′, antisense: 5′-ACGUGACACGUUCGGAGAATT-3′) were generated from Genepharma (Shanghai, China). These oligonucleotides (50 nM) and vectors (1 *μ*g) were transfected into cells at 60-70% confluence using 5 *μ*l Lipofectamine 2000 (Invitrogen, Carlsbad, CA, USA) for 6 h according to the instructions as previously reported with some modifications [16]. Cells were collected at 24 h after the transfection for subsequent experiments.

### 2.3. Quantitative Real-Time Polymerase Chain Reaction (qRT-PCR)

For detection of the abundances of CASC2 and miR-155 in PTC tissues and cells, Trizol reagent (Thermo Fisher) was used for total RNA isolation. Then, 1 *μ*g RNA was reversely transcribed to cDNA using TaqMan microRNA reverse transcription kit or High-Capacity cDNA Reverse Transcription Kit (Thermo Fisher).The cDNA was mixed with SYBR Green Mix (Thermo Fisher) and specific primers and used for qRT-PCR on a 7900HT Fast RT-PCR. The amplification procedure was as follows: 95°C for 5 min and 40 cycles at 95°C for 10 s, 60°C for 30 s. The primers were listed as follows: CASC2 (forward, 5′-GGCTCACAAAGCCTAGGTTA-3′; reverse, 5′-CCTTGGATATTTCCAAGAGC-3′); miR-155 (forward, 5′-CCCCACAGTCTACTGTAAG-3′; reverse, 5′-GCATTGCCGATGGTACTGATT-3′). GAPDH (forward, 5′-AGAAGGCTGGGGCTCATTTG-3′; reverse, 5′-AGGGGCCATCCACAGTCTTC-3′) and U6 (forward, 5′-CTCGCTTCGGCAGCACA-3′; reverse, 5′-AACGCTTCACGAATTTGCGT-3′) were used as internal controls. The relative levelsof CASC2 and miR-155 were calculated by the 2^-*ΔΔ*Ct^ method [17].

### 2.4. MTT

1 × 10^4^ cells were seeded into 96-well plates and cultured for 0, 24, 48, or 72 h. At the ending time, 10 *μ*l MTT solution (Beyotime, Shanghai, China) was added to each well and incubated for 4 h. Subsequently, the medium was replaced with 150 *μ*l DMSO solution (Sigma, St. Louis, MO, USA) to dissolve the formed formazan. The absorbance was measured at 490 nm with a microplate reader.

### 2.5. Flow Cytometry

The treated sensitive or resistant cells were collected and resuspended in binding buffer and used for apoptosis detection using Annexin V-FITC/PI kit (Beyotime) according to the manufacturer's instructions. The apoptotic cells were analyzed by flow cytometry, and apoptotic rate was expressed as percentage of cells at early apoptosis (Annexin V-FITC positive and PI negative) and late apoptosis (Annexin V-FITC positive and PI positive).

### 2.6. Western Blot

res-TPC-1 and res-IHH-4 cells were harvested and lysed in RIPA buffer (Beyotime). Cell lysates were quantified by BCA kit (Thermo Fisher) and separated by SDS-PAGE and then transferred to PVDF membranes (Millipore, Billerica, MA, USA). The membranes were blocked using specific blocking solution and then interacted with primary antibody anti-forkhead box O3 (FOXO3) (Cell Signaling Technology, Danvers, MA, USA) and the HRP-labeled secondary antibody IgG (Cell Signaling Technology). Anti-*β*-actin (Cell Signaling Technology) acted as a loading control. The ECL Western Blotting Substrate kit (Solarbio, Beijing, China) was used to develop the protein signals.

### 2.7. Luciferase Assay and RNA Immunoprecipitation (RIP)

starBase predicted the potential binding sites of CASC2 and miR-155. DIANA tools predicted the complementary sites of miR-155 and FOXO3. The sequences of CASC2 containing wild-type (AGCAUUA) or mutant (GAACGUC) binding sites of miR-155 were inserted into pmirGLO vectors (Promega, Madison, WI, USA) to generate corresponding luciferase reporter vectors CASC2-WT and CASC2-MUT, respectively. res-TPC-1 and res-IHH-4 cells cotransfected with luciferase constructs and miR-155 mimic ormiR-NC were used for luciferase assay with a luciferase reporter assay kit (Promega) after the transfection for 24 h.

res-TPC-1 and res-IHH-4 cells transfected with miR-155 mimic or miR-NC were also used for RIP assay with the Magna RNA Immunoprecipitation Kit (Millipore) according to the manufacturer's instructions. The anti-Ago2 (ab32381, Abcam, Cambridge, MA, USA) and IgG (AP112, Sigma) were used. The level of CASC2 enriched in complex by RIP was detected by qRT-PCR.

### 2.8. *In Vivo* Tumor Growth Assay

BALB/c nude mice (six weeks old, male, 18-22 g) were obtained from Vital River Laboratory Animal Technology (Beijing, China) and housed under standard conditions. For tumor growth assay, 1 × 10^6^ res-TPC-1 cells (empty group) or res-TPC-1 cells transfected with a control lentivirus (lenti-NC group) or a recombinant lentivirus-expressing CASC2 (lenti-CASC2 group) were subcutaneously injected into the back of mice. After inoculation, tumor volume was monitored and calculated every week for a total of five weeks according to the formula (length × width^2^)/2. At five weeks after injection, mice were euthanized by intraperitoneal phenobarbital injection (150 mg/kg; Guangdong Bangmin Pharmaceutical Co., Ltd., Guangdong, China), and tumors were removed for weight and qRT-PCR assay. The animal work was performed at Xinyang Vocational and Technical College and with the approval of Animal Ethics Committee of our college.

### 2.9. Statistical Analysis

Data of three independent experiments in graph were expressed as mean ± standard deviation (S.D.) and investigated by Student's *t* test or ANOVA followed by Tukey's post hoc test, supported by GraphPad Prism 7 software (La Jolla, CA, USA). Overall survival of patients with PTC after ^131^I therapy was analyzed by Kaplan-Meier plot and log-rank test. The association between CASC2 level and clinical features of PTC patients was analyzed by the *χ*^2^ test. *P* < 0.05 was considered statistically significant (^∗^*P* < 0.05, ^∗∗^*P* < 0.01, and ^∗∗∗^*P* < 0.001).

## 3. Results

### 3.1. The Expression of CASC2 Is Reduced in PTC

**Figure 1:**
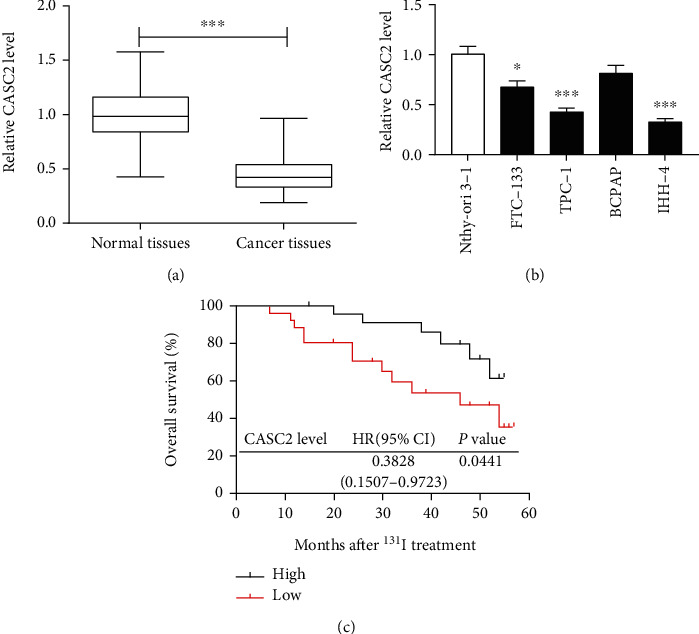
CASC2 expression is decreased in papillary thyroid cancer. (a) qRT-PCR was performed to measure the expression of CASC2 in papillary thyroid cancer tissues and adjacent normal tissues (*n* = 46). (b) qRT-PCR assay detected the level of CASC2 in thyroid cancer cells. (c) Overall survival of patients after ^131^I therapy was analyzed in low and high CASC2 level groups. ^∗^*P* < 0.05, ^∗∗^*P* < 0.01, and ^∗∗∗^*P* < 0.001.

To explore the role of CASC2 in PTC, we first measured its expression in 46 cancer tissues. As shown in Figure 1(a), the level of CASC2 was significantly decreased in cancer tissues in comparison to that in the matched normal tissues. Moreover, the patients were divided into high (*n* = 21) or low (*n* = 25) CASC2 level group according to its mean abundance in cancer tissues.  Table 1 summarizes that low expression of CASC2 was associated with pathological stage, tumor size, and lymph node metastasis (*P* < 0.05) but not with age and gender (*P* > 0.05) of patients. Furthermore, CASC2 was obviously lowly expressed in thyroid cancer cells (FTC-133, TPC-1, and IHH-4) when compared with the control group (Nthy-ori 3-1) (Figure 1(b)). In addition, another 50 patients with treatment of ^131^I were divided into two groups according to the median value of CASC2, and the patients with low expression of CASC2 (*n* = 25) exhibited poorer overall survival than those with high expression (*n* = 25) (hazard ratio (HR) = 0.3828; 95% confidence interval (CI) (0.1507-0.9723); *P* = 0.0441) (Figure 1(c)).

### 3.2. The Construction of ^131^I Tolerance of PTC Cells

**Figure 2:**
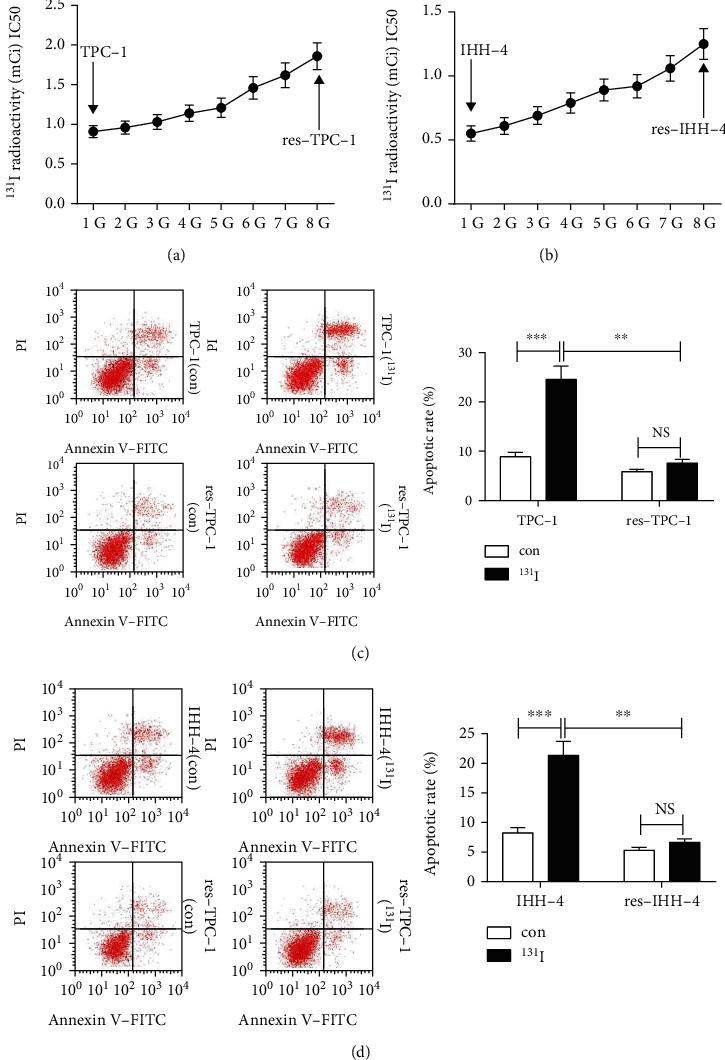
Continuous exposure to ^131^I induces tolerance of papillary thyroid cancer cells. (a, b) TPC-1 and IHH-4 cells were treated with ^131^I, and ^131^I-resistant cells (res-TPC-1 and res-IHH-4) were obtained after 8 generations. The IC50 of ^131^I was analyzed by MTT assay. (c, d) Flow cytometry was conducted to measure cell apoptosis in TPC-1, res-TPC-1, IHH-4, and res-IHH-4 cells after treatment of the indicated dose of ^131^I for 12 h. ^∗∗^*P* < 0.01 and ^∗∗∗^*P* < 0.001; NS: not significant.

To explore the pathogenesis of ^131^I resistance in PTC, the ^131^I-resistant cells were constructed by using TPC-1 and IHH-4 cells with relative lower level of CASC2 through continuous exposure to ^131^I. As displayed in Figures 2(a) and 2(b), the corresponding resistant cells (res-TPC-1 and res-IHH-4) were generated after passage for 8 generations (G); the IC50 of ^131^I was increased from 1 to 1.66 mCi in TPC-1 cells and 0.5 to 1.1 mCi in IHH-4 cells. Furthermore, the resistant res-TPC-1 and res-IHH-4 avoided to cell apoptosis in corresponding sensitive cells induced by exposure to ^131^I (Figures 2(c) and 2(d)).

### 3.3. Knockdown of CASC2 Decreases ^131^I Sensitivity in PTC Cells

**Figure 3:**
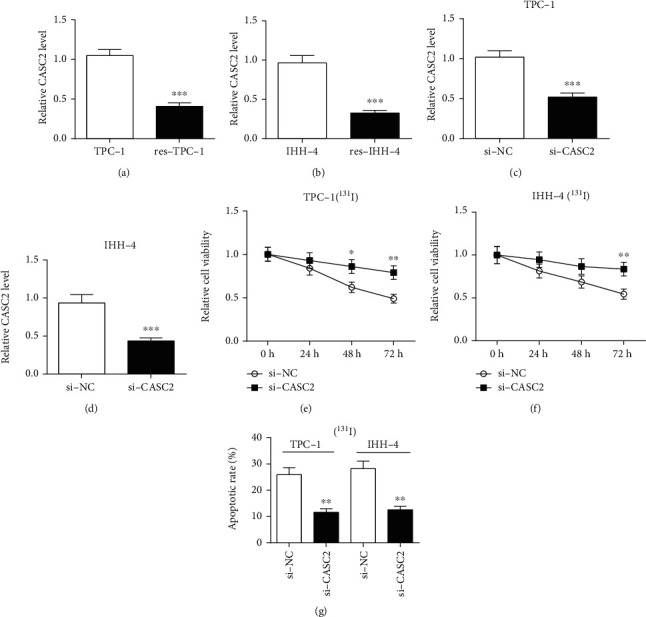
Knockdown of CASC2 decreases ^131^I sensitivity in sensitive papillary thyroid cancer cells. (a, b) qRT-PCR detected the level of CASC2 in TPC-1, res-TPC-1, IHH-4, and res-IHH-4 cells. (c, d) qRT-PCR measured the abundance change of CASC2 in TPC-1 and IHH-4 cells after transfection of si-CASC2 or si-NC. (e, f) Cell viability and (g) apoptosis in TPC-1 and IHH-4 cells transfected with si-CASC2 or si-NC were analyzed after treatment of the indicated dose of ^131^I for 12 h. ^∗^*P* < 0.05, ^∗∗^*P* < 0.01, and ^∗∗∗^*P* < 0.001.

As displayed in Figures 3(a) and 3(b), the expression of CASC2 was greatly reduced in res-TPC-1 and res-IHH-4 cells compared with the corresponding TPC-1 and IHH-4 cells. To investigate the effect of CASC2 on ^131^I sensitivity in sensitive PTC cells, si-CASC2 was used to knock down the abundance of CASC2 in TPC-1 and IHH-4 cells, which was confirmed in Figures 3(c) and 3(d). Furthermore, silencing CASC2 promoted cell viability induced by ^131^I in the two cell lines compared with treatment of si-NC (Figures 3(e) and 3(f)). In addition, knockdown of CASC2 inhibited ^131^I-caused apoptosis in TPC-1 and IHH-4 cells (Figure 3(g)).

### 3.4. Overexpression of CASC2 Improves ^131^I Sensitivity in Resistant PTC Cells

**Figure 4:**
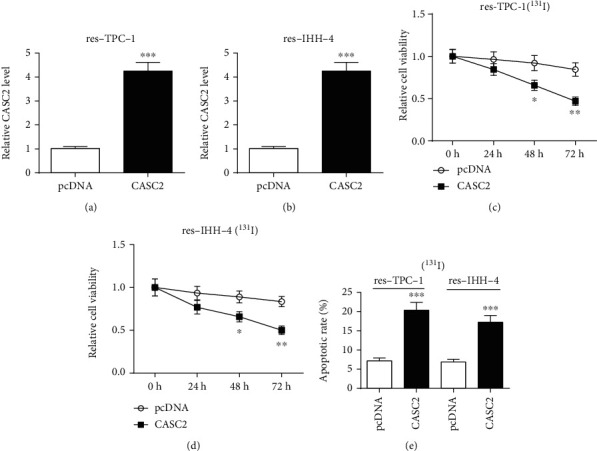
Overexpression of CASC2 inhibits ^131^I resistance in resistant papillary thyroid cancer cells. (a, b) qRT-PCR was used to measure the expression change of CASC2 in res-TPC-1 and res-IHH-4 cells after transfection of CASC2 overexpression vector or pcDNA. (c, d) Cell viability and (e) apoptosis in res-TPC-1 and res-IHH-4 cells transfected with CASC2 overexpression vector or pcDNA were determined after treatment of the indicated dose of ^131^I for 12 h. ^∗^*P* < 0.05, ^∗∗^*P* < 0.01, and ^∗∗∗^*P* < 0.001.

To evaluate the function of CASC2 on ^131^I sensitivity in resistant PTC cells, the abundance of CASC2 was overexpressed in res-TPC-1 and res-IHH-4 cells through transfection of CASC2 overexpression vector (Figures 4(a) and 4(b)). Furthermore, upregulation of CASC2 exacerbated ^131^I-induced viability suppression in res-TPC-1 and res-IHH-4 cells (Figures 4(c) and 4(d)). Moreover, the addition of CASC2 enhanced the apoptotic rate of res-TPC-1 and res-IHH-4 cells with exposure to ^131^I (Figure 4(e)).

### 3.5. miR-155 Is a Target of CASC2

**Figure 5:**
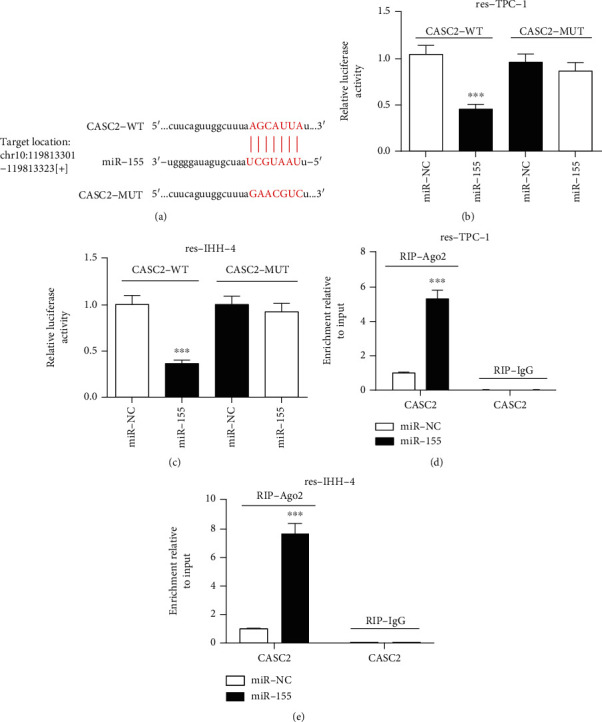
CASC2 is a sponge of miR-155. (a) The binding sites of CASC2 and miR-155 were provided by starBase. (b, c) Luciferase activity was analyzed in res-TPC-1 and res-IHH-4 cells cotransfected with CASC2-WT or CASC2-MUT and miR-155 mimic or miR-NC. (d, e) RIP assay was performed in res-TPC-1 and res-IHH-4 cells transfected with miR-155 mimic or miR-NC, and enriched CASC2 level was measured by qRT-PCR. ^∗∗∗^*P* < 0.001.

lncRNA could serve as miRNA sponge in cancer progression. The analysis of starBase online predicted the binding sites of miR-155 and CASC2 at chr10: 119813301-119813323 (Figure 5(a)). In order to validate the target association between CASC2 and miR-155, luciferase assay and RIP assay were performed in res-TPC-1 and res-IHH-4 cells. As described in Figures 5(b) and 5(c), overexpression of miR-155 resulted in more than 55% reduction of luciferase activity in res-TPC-1 and res-IHH-4 cells, respectively, in the CASC2-WT group, while the activity was not changed in the CASC2-MUT group. Moreover, overexpression of miR-155 induced 5.1-fold and 7.7-fold elevation in enrichment level of CASC2 by Ago2 RIP, but IgG failed to display the enrichment (Figures 5(d) and 5(e)).

### 3.6. CASC2 Enhances ^131^I Sensitivity in Resistant PTC Cells by Regulating miR-155

**Figure 6:**
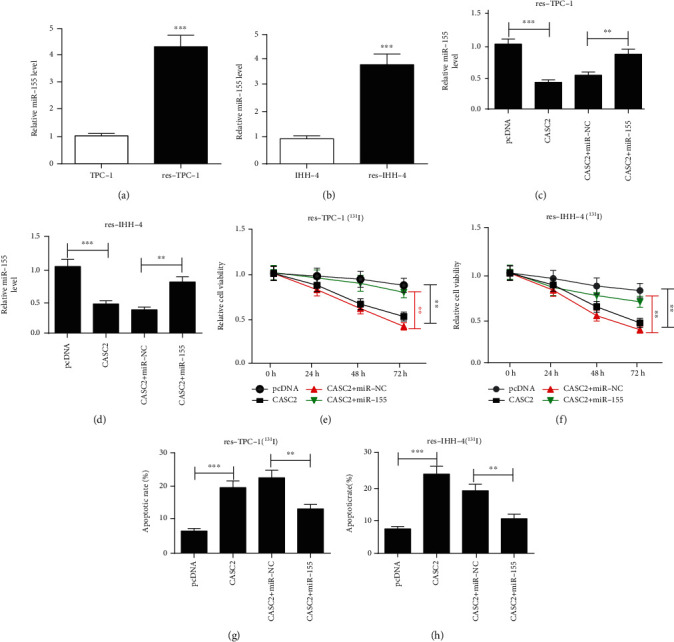
miR-155 attenuates CASC2-mediated ^131^I resistance inhibition in resistant papillary thyroid cancer cells. (a, b) qRT-PCR detected the level of miR-155 in TPC-1, res-TPC-1, IHH-4, and res-IHH-4 cells. (c, d) qRT-PCR was carried out to measure the expression change of miR-155 in res-TPC-1 and res-IHH-4 cells after transfection of pcDNA, CASC2 overexpression vector, CASC2 overexpression vector+miR-NC, or miR-155 mimic. (e, f) Cell viability and (g, h) apoptosis were examined in res-TPC-1 and res-IHH-4 cells transfected with pcDNA, CASC2 overexpression vector, CASC2 overexpression vector+miR-NC, or miR-155 mimic after exposure to the indicated dose of ^131^I for 12 h. ^∗∗^*P* < 0.01 and ^∗∗∗^*P* < 0.001.

As shown in Figures 6(a) and 6(b), the expression level of miR-155 was significantly elevated in resistant cells in comparison to sensitive cells. To explore whether miR-155 is involved in CASC2-mediated ^131^I sensitivity in PTC cells, res-TPC-1 and res-IHH-4 cells were transfected with pcDNA, CASC2 overexpression vector, CASC2 overexpression vector+miR-NC, or miR-155 mimic. As displayed in Figures 6(c) and 6(d), the abundance of miR-155 was evidently reduced by CASC2 overexpression, which was restored by transfection of miR-155 mimic. Furthermore, the cell viability inhibited by CASC2 overexpression was obviously increased by upregulation of miR-155 in res-TPC-1 and res-IHH-4 cells treated with ^131^I (Figures 6(e) and 6(f)). Moreover, the promotion role of CASC2 on apoptosis was weakened by introduction of miR-155 in the two cell lines (Figures 6(g) and 6(h)). Additionally, the targets of miR-155 with the miTG score > 0.7 were predicted via DIANA tools, and 10 targets relevant to radioresistance were selected (Supplementary Figure 1A). FOXO3 was one important target of miR-155, and the target site is shown in Supplementary Figure 1B. FOXO3 protein level was negatively regulated via miR-155 in res-TPC-1 and res-IHH-4 cells (Supplementary Figures 1C and 1D). Moreover, by phylogenetic assay using the UCSC Genome Browser (http://genome.ucsc.edu/), miR-155 binding site across species is highly conserved among mammals (Supplementary Figure 1E).

### 3.7. CASC2 Decreases ^131^I-Resistant PTC Cell Growth *In Vivo*

**Figure 7:**
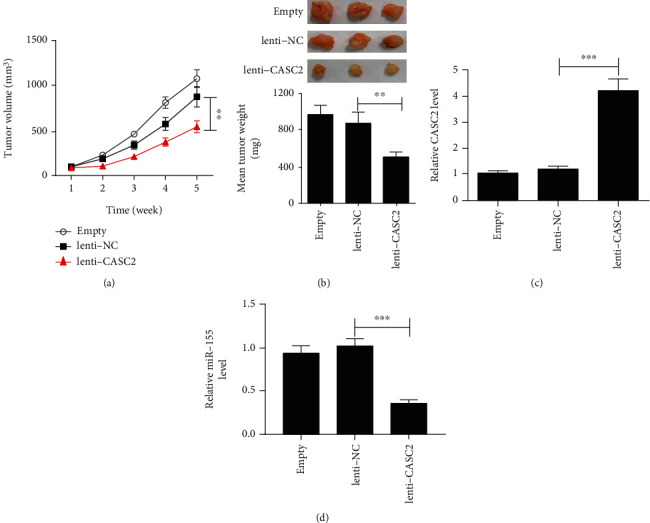
CASC2 reduces the growth of ^131^I-resistant cells in vivo. (a) Tumor volume was measured and calculated every week. (b) Tumors were weighed after 5 weeks after implantation. (c, d) qRT-PCR was performed to test the expression change of CASC2 and miR-155. ^∗∗^*P* < 0.01 and ^∗∗∗^*P* < 0.001.

To explore whether CASC2 affected the growth of ^131^I-resistant cells *in vivo*, res-TPC-1 cells or res-TPC-1 cells stably transfected with lenti-NC or lenti-CASC2 were injected into the mice. Tumor volumes were examined every week, and xenografts were removed at the fifth week. As presented in Figure 7(a), tumor volumes kept increasing after injection. Compared with the empty and lenti-NC groups, overexpression of CASC2 decreased tumor volumes at each time point. Moreover, tumor weight was also significantly reduced in the CASC2-expressing group relative to the empty and lenti-NC groups (Figure 7(b)). The results of qRT-PCR assay revealed that stable transfection of lenti-CASC2 markedly increased the level of CASC2, but decreased miR-155 level in tumor tissues (Figures 7(c) and 7(d)).

## 4. Discussion

In this study, we found that CASC2 was lowly expressed in PTC tissues and cells and indicated poor prognosis of patients, which is also in agreement with former efforts [14, 15, 18]. ^131^I therapy is a common strategy for the treatment of thyroid cancer. However, the interaction between CASC2 and ^131^I sensitivity is far from being understood. Our research was the first to study that CASC2 could increase ^131^I sensitivity to PTC and explored the potential targeted miRNA.

To investigate the pathogenesis of ^131^I resistance, the resistant cells were first constructed based on the sensitive cells. After 8 generations, the constructed resistant cells res-TPC-1 and res-IHH-4 avoided the ^131^I-induced apoptosis. This validated the successful construction of ^131^I-resistant cells. Then, the qRT-PCR results of reduced CASC2 in resistant cells revealed that low expression of CASC2 might be associated with ^131^I resistance. A former work suggested the antiproliferation and proapoptosis role of CASC2 in PTC cells [15]. Similarly, by loss-of-function and gain-of-function experiments, we also found that CASC2 silence increased cell viability but decreased apoptosis and DNA damage in PTC cells exposed by ^131^I, while CASC2 addition played an opposite effect. *In vivo* experiments also indicated that abundance of CASC2 inhibited the growth in ^131^I-resistant cell-formed tumor, which indicated that CASC2 could act as a therapeutic target for ^131^I therapy of PTC.

Previous studies indicated that CASC2 could serve as a competing endogenous RNA (ceRNA) or miRNA sponge in the development of cancers [12, 19]. Accruing reports demonstrated that miR-155 is an oncogenic miRNA involved in drug resistance and radioresistance in human cancers [20–22]. Furthermore, it is suggested that miR-155 expression was increased in papillary thyroid carcinoma and its overexpression promoted cell proliferation by regulating adenomatous polyposis coli and Wnt/*β*-catenin signaling [23–25]. In this study, we first confirmed the association between CASC2 and miR-155 in PTC cells by luciferase assay and RIP. Moreover, we found that the expression of miR-155 was higher in resistant cells than in sensitive cells, uncovering that upregulation of miR-155 might contribute to ^131^I resistance. By rescue experiments, results showed that miR-155 reversed CASC2-mediated promotion of ^131^I sensitivity, reflecting that CASC2 could regulate ^131^I sensitivity by sponging miR-155 in PTC. In order to better understand that ceRNA mechanism allows CASC2 in ^131^I sensitivity, the target of miR-155 might be helpful. We used DIANA tools to predict 10 targets of miR-155, which were reported to be relevant to radioresistance [21, 26–34]. As one of the predicted targets, forkhead box O3 (FOXO3) was involved in miR-155-mediated regulation of radio- or chemoresistance in thyrocytes [35, 36]. This study also confirmed that FOXO3 was targeted and negatively regulated via miR-155 in res-TPC-1 and res-IHH-4 cells. Hence, we hypothesized that FOXO3 might be responsible for CASC2 in ^131^I sensitivity to PTC by the crosstalk of miR-155, which would be further confirmed in the future.

In conclusion, low expression of CASC2 was showed in PTC and indicated poor outcomes of patients. CASC2 knockdown reduced ^131^I sensitivity in PTC cells, while its overexpression increased the sensitivity, possibly by regulation of miR-155. This study indicates a new mechanism for the development of ^131^I resistance and provides a novel target for the treatment of PTC.

## Figures and Tables

**Table 1:** The association between clinicopathologic features of patients and CASC2 level.

Clinicopathologic features	*N* (%)	Relative CASC2 level		*P* value
		Low (%)	High (%)	
Total cases	46 (100.0)	25 (54.3)	21 (45.7)	
Age (years)				*P* > 0.05
≥55	26 (56.5)	14 (53.8)	12 (46.2)	
<55	20 (43.5)	11 (55.0)	9 (45.0)	
Gender				*P* > 0.05
Male	28 (60.9)	15 (53.6)	13 (46.4)	
Female	18 (39.1)	10 (55.6)	8 (45.4)	
Pathological stage				*P* < 0.05
I-II	25 (54.3)	10 (40.0)	15 (60.0)	
III-IV	21 (45.7)	15 (71.4)	6 (28.6)	
Tumor size (cm)				*P* < 0.05
≥2.5	21 (45.7)	15 (71.4)	6 (28.6)	
<2.5	25 (54.3)	10 (40.0)	15 (60.0)	
Lymph node metastasis				*P* < 0.05
No	26 (56.5)	10 (38.5)	16 (61.5)	
Yes	20 (43.5)	15 (75.0)	5 (25.0)	

## Data Availability

The data displayed in this manuscript is available from the corresponding author upon reasonable request.

## Supplementary Materials

Supplementary Materials Supplementary Figure 1: the regulation of miR-155 on FOXO3. (A) The targets of miR-155 were predicted via DIANA tools, and 10 genes associated with radioresistance were shown. (B) The binding sites of miR-155 and FOXO3. (C and D) The effect of miR-155 on FOXO3 protein in res-TPC-1 and res-IHH-4 cells transfected with miR-155 mimic, miR-NC, anti-miR-NC, or anti-miR-155. (E) Multispecies comparisons of miR-155 using UCSC genome browser.
