# Supplementary material for: lncRNA CASC2 Enhances 131I Sensitivity in Papillary Thyroid Cancer by Sponging miR-155
Source: Biomed Res Int. 2020 Oct 19;2020:7183629. doi: 10.1155/2020/7183629 (PMC7591961; doi:10.1155/2020/7183629)

Supplementary Figure 1. The regulation of miR-155 on FOXO3. (A) The targets of miR-155 were predicted via DIANA tools, and 10 genes associated with radioresistance were shown. (B) The binding sites of miR-155 and FOXO3. (C and D) The effect of miR-155 on FOXO3 protein in res-TPC-1 and res-IHH-4 cells transfected with miR-155 mimic, miR-NC, anti-miR-NC or anti-miR-155. (E) Multi-species comparisons of miR-155 usingUCSC genome browser.

A

	Ensembl Gene Id	miRNA name	miTG score
1	ENSG00000166483 (WEE1)	hsa-miR-155-5p	0.996432586428355
2	ENSG00000135018 (UBQLN1)	hsa-miR-155-5p	0.969694036032594
3	ENSG00000185591 (SP1)	hsa-miR-155-5p	0.922925902097959
4	ENSG00000167244 (IGF2)	hsa-miR-155-5p	0.875189806230118
5	ENSG00000145779 (TNFAIP8)	hsa-miR-155-5p	0.854483998534067
6	ENSG00000118689 (FOXO3)	hsa-miR-155-5p	0.786444364409102
7	ENSG00000070950 (RAD18)	hsa-miR-155-5p	0.754074300928784
8	ENSG00000150593 (PDCD4)	hsa-miR-155-5p	0.750107301909601
9	ENSG00000096717 (SIRT1)	hsa-miR-155-5p	0.71820070107276
10	ENSG00000168785 (TSPAN5)	hsa-miR-155-5p	0.708549654957857

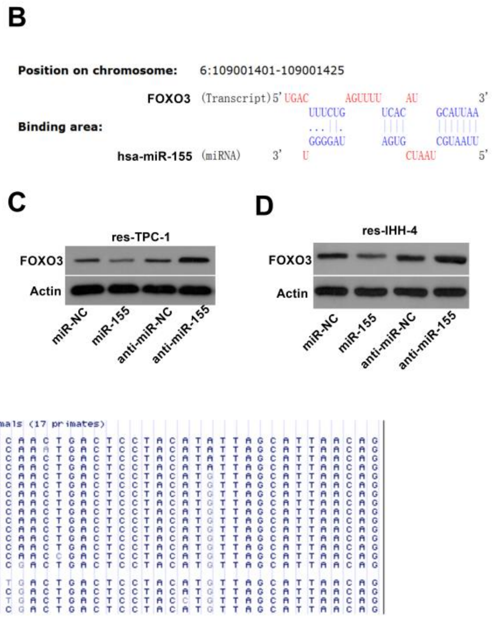

Supplement: Supplementary Materials — Supplementary Figure 1: the regulation of miR-155 on FOXO3. (A) The targets of miR-155 were predicted via DIANA tools, and 10 genes associated with radioresistance were shown. (B) The binding sites of miR-155 and FOXO3. (C and D) The effect of miR-155 on FOXO3 protein in res-TPC-1 and res-IHH-4 cells transfected with miR-155 mimic, miR-NC, anti-miR-NC, or anti-miR-155. (E) Multispecies comparisons of miR-155 using UCSC genome browser. [file 7183629.f1.pdf]
